# Incrimination of *Aedes (Stegomyia) hensilli* Farner as an Epidemic Vector of Chikungunya Virus on Yap Island, Federated States of Micronesia, 2013

**DOI:** 10.4269/ajtmh.14-0374

**Published:** 2015-02-04

**Authors:** Harry M. Savage, Jeremy P. Ledermann, Laurence Yug, Kristen L. Burkhalter, Maria Marfel, W. Thane Hancock

**Affiliations:** Centers for Disease Control and Prevention, Fort Collins, Colorado; Yap Hospital and Yap State Department of Health, Yap, Federated States of Micronesia

## Abstract

Two species of *Aedes (Stegomyia)* were collected in response to the first chikungunya virus (CHIKV) outbreak on Yap Island: the native species *Ae. hensilli* Farner and the introduced species *Ae. aegypti* (L.). Fourteen CHIKV-positive mosquito pools were detected. Six pools were composed of female *Ae. hensilli*, six pools were composed of female *Ae. aegypti*, one pool was composed of male *Ae. hensilli*, and one pool contained female specimens identified as *Ae. (Stg.)* spp. Infection rates were not significantly different between female *Ae. hensilli* and *Ae. aegypti*. The occurrence of human cases in all areas of Yap Island and the greater number of sites that yielded virus from *Ae. hensilli* combined with the ubiquitous distribution of this species incriminate *Ae. hensilli* as the most important vector of CHIKV during the outbreak. Phylogenic analysis shows that virus strains on Yap are members of the Asia lineage and closely related to strains currently circulating in the Caribbean.

## Introduction

Chikungunya virus (CHIKV) is a member of the genus *Alphavirus* within the family *Togaviridae*.^1^,^2^ The virus is endemic to Africa, India, and Asia. Phylogenetic analysis has shown that CHIKV form three major lineages, which represent adaption to regional conditions: an East, Central, South African (ECSA) lineage, a West African (WA) lineage, and an Asian lineage. Recently, international commerce and tourism have resulted in the introduction of viruses, particularly members of the ECSA and Asian lineages, into new geographic areas, resulting in epidemic outbreaks.^3^–^6^

The disease associated with CHIKV was first described during an outbreak in Tanzania that occurred from 1952 to 1953. Virus isolations from field-collected mosquitoes and experimental transmission studies established *Aedes aegypti* (L.) as the primary epidemic vector of CHIKV.^7^ Subsequent studies in Africa detected a sylvan transmission cycle among wild primates and *Ae. (Stegomyia)* and *Ae. (Diceromyia)* mosquitoes including *Ae. (Stg.) africanus* (Theobald), *Ae. (Stg.) luteocephalus* (Newstead), and *Ae. (Dic.) furcifer* (Edwards), but *Ae. aegypti* remained the primary vector in early urban epidemics in both Africa and Asia.^1^ Experimental vector competence studies provided additional support for *Ae. furcifer* as an epidemic vector and implicated *Mansonia africana* (Theobald) as a potential vector in southern Africa.^8^ In 2004, CHIKV spread from Kenya onto the islands of the Indian Ocean, including Reunion Island and beyond.^4^,^5^ The epidemic on Reunion Island was unusual, because the epidemic vector was *Ae. (Stg.) albopictus* (Skuse); and subsequent vector competence studies showed that a single mutation, E1-A226V, enhanced virus replication and transmission in *Ae. albopictus*.^9^,^10^ CHIKV continued to spread through infected human travelers, and autochthonous cases were reported in temperate Italy in 2007.^3^ Entomological studies in Italy also showed that *Ae. albopictus* was the vector.^11^

On October 19, 2013, the Yap State Department of Health Services of the Federated States of Micronesia (FSM) contacted the Centers for Disease Control and Prevention (CDC) concerning an outbreak of unexplained illness on Yap characterized by acute onset of fever, arthralgia, and rash (Pastula D and Hancock WT, personal communication). On October 30, the Arboviral Diseases Branch (ADB) diagnostic laboratory of the Division of Vector-Borne Diseases (DVBD) of the CDC in Fort Collins, CO identified CHIKV in multiple patient serum samples sent from Yap. An entomological team from the ADB was dispatched in November and conducted entomological surveillance on Yap from November 13 to 18, 2013. Goals of the entomological surveillance team were to (1) implicate the vector(s) based on isolation of virus from field-collected specimens and (2) determine the infection rate (IR) in potential vector species.

## Materials and Methods

### Description of the study site.

**Figure 1.**
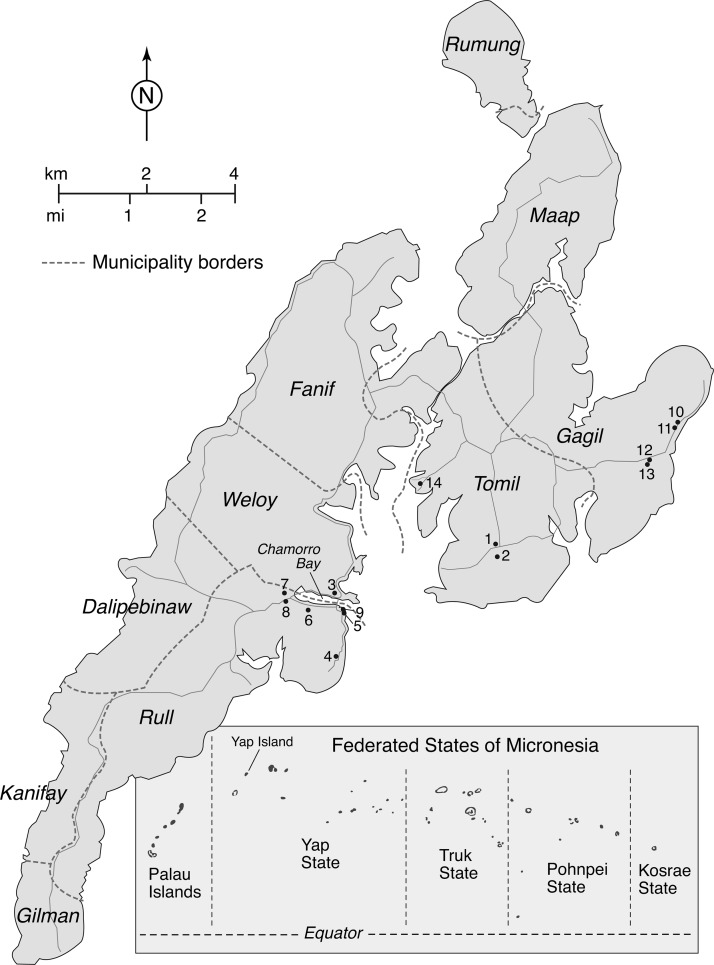
Map of Yap Island with 10 municipalities and 14 entomological collection sites labeled. Inset shows the location of Yap Island within the FSM and the location of Palau.

Yap State, comprising the main island group of Yap and 18 inhabited neighboring islands, is the westernmost state of the FSM (Figure 1). In the preliminary 2010 census data, the population of Yap State was 11,376 people, with approximately 7,370 people residing on Yap. Average annual household income for the FSM was $4,600 in 2000, with an average household size of seven persons (http://www.fsmgov.org/press/pr05300b.htm).

The main island group of Yap is composed of four closely associated islands, Marba, Gagil-Tomil, Maap, and Rumung, that lie within a fringing reef system. These four islands have a combined surface area of 100.4 km^2^ and are approximately 25 km long and 10 km wide at their widest point.^12^,^13^ The four main islands of Yap are the remnants of old, metamorphic, high volcanic islands, with a current maximum elevation of 174 m. The climate of Yap is characterized by constant warm temperatures, heavy rainfall, and high humidity. Mean annual rainfall is 3,028 mm. The driest months are February, March, and April, with an average monthly precipitation of less than 180 mm.^12^ The wettest season is July through October, when average monthly rainfall is 330 mm. Mean annual temperature is 27°C, with mean monthly variation of only 2°C. Daytime maximum and nighttime minimum temperatures differ by an average of 7°C. Mean relative humidity ranges from 79% to 85%.

The tropical island vegetation of Yap has been modified by agricultural practices. The main crops are taro, coconut, breadfruit, yams, bananas, tapioca, citrus, and betel nut. Livestock, including chickens and swine, are maintained by many residents. Currently, tourism and government employment play major roles in the local economy.

Yap is divided into 10 municipalities (Figure 1): Rumung, Maap, Gagil, Tomil, Fanif, Weloy, Dalipebinaw, Rull, Kanifay, and Gilman. The only urban area, Colonia, is located on Chamorro Bay and includes the port and government buildings.

### Mosquito collection sites and methods.

Mosquito collections were made on the main island of Yap, Yap State, FSM (Figure 1) from November 13 to 18, 2013. Collections were made at 14 sites: 12 suspect case-patient residences and 2 sites not associated with human disease; 11 of 12 suspect case-patient residences were single or extended family homes, whereas the remaining case-patient residence was located in an apartment complex (site 14). A list of names and residences for patients clinically diagnosed as having a recent or ongoing CHIKV infection by staff of the Yap State Hospital was provided to the entomological team. At each home, the entomological team verified that the patient resided at the residence and then obtained permission to collect mosquitoes. The 12 suspect case-patients residences were located in three municipalities (Figure 1): 4 residences in Gagil (sites 10–13), 2 residences in Tomil (sites 2 and 14), and 6 residences in Rull (sites 4–9). In addition, collections were made at two sites not associated with human disease: the Division of Public Safety (Police Department and jail), which is site 3 and located in Colonia (Weloy), and the Tomil Community Health Center (site 1).

Adult mosquito collections were made at all 14 sites with 1 to 2 BG-Sentinel traps baited with BG-Lure and octenol (BioQuip, Rancho Dominguez, CA). Traps were run for 24 hours, and collection bags were changed each morning. At selected sites (sites 4, 6–9, 11, and 13), adult mosquitoes were also collected with a large aspirator (InsectaZooka; BioQuip). Aspirator collections were brief (5–20 minutes) and focused on resting sites in and around case-patient houses.

Collection bags from BG traps and aspirator cups were labeled each morning, placed on gel packs in a cooler, and returned to the field laboratory. Mosquitoes were removed from other taxa and debris with the aid of a dissecting microscope and placed in labeled cryotubes. Specimens in cryotubes were maintained on Yap at −20°C and shipped on frozen gel packs to Guam, where they were transferred to a −70°C ultra-low freezer. On Guam, cryotubes were repackaged and shipped on dry ice to the CDC in Fort Collins, CO.

In addition, 16 mosquito ovitraps (black plastic cups) were placed at four sites on Yap. Sites included two suspect case-patient residences (sites 3 and 9), grounds of the Environmental Protection Agency in Colonia (near 09°31.003′ N, 138°07.241′ E), and a forested site in Fanif municipality (09°33.930′ N, 138°07.609′ E). Papers from ovitraps were collected, dried, and packaged for shipment to the CDC in Fort Collins, CO, where the eggs were hatched and larvae were reared to the adult stage for identification.

### Mosquito processing and initial virus testing.

All mosquitoes were identified on refrigerated chill tables using a dissecting microscope and taxonomic references,^14^–^16^ and they were grouped into pools of 50 or less based on species, sex, site, and date.

The mosquito pools were placed into 2-mL tubes (Axygen Scientific, Union City, CA) with a BB and 1.75 mL bovine albumin-1 (BA-1) media, which consisted of Medium 199 with Hank's salts (M199-H; Sigma-Aldrich, St. Louis, MO), 0.05 M Tris buffer (pH 7.5; Invitrogen, Carlsbad, CA), 1% bovine serum albumin (Probumin, pH 7.0; Millipore, Billerica, MA), 2 mM l-glutamine (Invitrogen), 4.2 mM sodium bicarbonate (Invitrogen), 100 U/mL penicillin (Invitrogen), 100 μg/mL streptomycin (Invitrogen), and 1 μg/mL amphotericin B (Fungizone; Sigma-Aldrich). Mosquitoes were homogenized with a Mixer Mill (Qiagen, Valencia, CA) set at 4 minutes and 25 seconds. The homogenized mosquitoes were then centrifuged at 10,000 × *g* for 4 minutes. A 125-μL aliquot of the supernatant of each pool was removed and placed in an identically labeled Eppendorf tube for RNA extraction and testing with a real-time reverse transcriptase polymerase chain reaction (RT-PCR) assay (see below). On the same day, six-well plates (Corning, Corning, NY) containing a monolayer of Vero cells were inoculated with 250 μL mosquito pool homogenate filtered through a 0.20-μM syringe filter (Pall, Port Washington, NY) and placed into an incubator set at 37°C and 5% CO_2_. After 1 hour, 2 mL Dulbecco's minimal essential medium (DMEM; Gibco, Carlsbad, CA) supplemented with 10% fetal bovine serum (FBS), 100 U/mL penicillin and streptomycin, and 1 U/mL fungizone and gentamycin was added to each well. The cell and homogenized mosquito mixture were then monitored daily for cytopathic effect (CPE) for 7 days. Media from those wells presenting signs of CPE were harvested and placed at −70°C.

To test samples with a second methodology and detect low-titered samples, we also screened an aliquot of each mosquito pool for viral RNA using a real-time RT-PCR assay similar to that described previously,^17^ except with the use of a new primer/probe set (3855/3957c/3886 FAM) designed to detect Asian and ECSA genotypes of CHIKV (Lanciotti RS, personal communication). Sequence information is as follows: CHIKV 3855, GAGCATACGGTTACGCAGATAG; CHIKV 3957c–a, TACTGGTGATACATGGTGGTTTC; CHIKV 3957c–b, TGCTGGTGACACATGGTGGTTTC; CHIKV 3886 FAM-a, ACGAGTAATCTGCGTACTGGGACGTA; and CHIKV 3886 FAM-b, ACGAGTCATCTGCGTATTGGGACGCA. Primer 3957c and probe 3886 FAM are produced by first reconstituting the individual a and b primers to 100 μM and the individual a and b probes to 25 μM and then mixing a and b in equal volumes to produce the mixed working stocks. Viral RNA was extracted from a 100-μL aliquot of supernatant taken from each mosquito pool using the Qiagen QIAamp Virus BioRobot 9604 Kit (Qiagen) on a Qiagen BioRobot Universal platform according to the manufacturer's protocol. Real-time RT-PCR was used to screen for CHIKV by adding 10 μL of each extracted RNA to 50 pmol primers and 10 pmol probes specific to the non-structural (nsP2) region of the CHIKV genome as described above and reagents from Qiagen's Quantitect Probe RT-PCR Kit according to the manufacturer's instructions. Reactions were conducted on the CFX96 Touch Real-Time PCR Detection System (Bio-Rad, Hercules, CA) according to the manufacturer's recommended cycling conditions.

### Virus confirmation and sequencing.

From each mosquito pool that produced CPE in cell culture, viral RNA was isolated from the first Vero (V1) harvest using the QIAamp Viral RNA Kit according to the manufacturer's protocol (Qiagen). Total RNA was extracted from 140 μL cell supernatant and eluted from the kit columns in 60 μL elution buffer. Traditional RT-PCR assays were used to detect viral nucleic acid and for Sanger sequencing. For virus identification, a 5-μL aliquot of purified RNA was subjected to amplification using the OneStep RT-PCR Kit protocol (Qiagen) and combined with the flavivirus^18^ or alphavirus group primers^19^ or those specifically designed for the CHIKV genome: CHIKV 301, CAGGAAGTACCACTGCGTCTGCC^4^ and CHIKV 1303, CCCCAGGAGTTTTTCATCTTCCATGTC (Ledermann JP and Powers AM, personal communication). The manufacturer's protocol was followed without modifications. The reactions were analyzed by gel electrophoresis, and correctly sized products were extracted and cleaned using the MinElute Gel Extraction Kit (Qiagen) and subsequently sequenced.

Two mosquito isolates (one from each vector species) were selected for full-length sequencing. The 5′ and 3′ genome termini were obtained using the 5′/3′ RACE Kit protocol following the manufacturer's recommendations (Roche, Indianapolis, IN). Sequencing reactions were performed on the PCR products using the Big Dye v3.1 Kit on an ABI 3130xl genetic analyzer (Applied Biosystems, Grand Island, NY) and CHIKV-specific primers (provided on request). Sequencing chromatograms were analyzed for sequence quality and assembly using the Lasergene 9 Core suite software (DNASTAR, Madison, WI).

### Phylogenetic analysis.

Trees were constructed based on 33 published nucleotide sequences representing several members from each CHIKV genotype. Sequences were aligned using the ClustalW software algorithm, and the phylogenic trees were constructed under partial deletion of gaps. The evolutionary history was inferred using the maximum likelihood method based on the Tamura–Nei model with 1,000 bootstrap iterations, and a combination of maximum parsimony and MIONJ method with MCL distance matrix was used (MEGA 5.05).^20^,^21^ There were 11,244 positions in the final dataset.

## Results

### Entomological collections and IRs.

Entomological collections made with BG traps and aspirators from November 13 to 18, 2013 at 14 sites (including 12 suspect case-patient houses) resulted in the collection of 1,660 mosquito specimens representing seven taxa (Table 1). Males were more frequently collected than females and represented 58% of collections. *Culex (Cux.) quinquefasciatus* Say was the most frequently collected species, being detected at all sites except case-patient residences 5, 10, and 13 (Figure 1). However one site, the Tomil Community Health Center (site 1), accounted for the majority of specimens (701; 72.9%), which apparently resulted from localized production from a septic system.

Two species of *Ae. (Stg.)* were represented in collections (Table 1). The native species *Ae. (Stg.) hensilli* Farner was the second most common species represented in our collections (487 [29.3%] specimens), whereas the introduced species *Ae. (Stg.) aegypti* was the third most frequently collected species (193 [11.6%] specimens). *Ae. hensilli* is nearly ubiquitous on Yap (Savage HM and Godsey M, unpublished data) and was collected at 11 of 14 sites (78.6%; all sites except 5, 9, and 14) (Figure 1). In contrast, we only detected *Ae. aegypti* in collections from six (42.9%) sites (sites 3, 6, 7, 8, 9, and 14). The first five of the *Ae. aegypti*-positive sites were located in or near Colonia, whereas site 14 was an apartment complex housing persons previously displaced from other islands by a natural disaster (Figure 1). Four sites (sites 3 and 6–8) located in Colonia and nearby developed areas of Rull yielded both species.

Homogenates from all 238 mosquito pools were inoculated onto Vero cells. Thirteen pools produced CPE in cell culture, and viral RNA isolated from V1 harvests was subsequently identified as CHIKV by RT-PCR (Table 2). Testing all pools by real-time RT-PCR confirmed these results and detected one additional viral RNA-positive pool, YAP-13-2169 (Table 2). Retesting of pool YAP-13-2169 failed to detect viable virus.

Thirteen of the CHIKV-positive pools were composed of female *Ae. (Stg.)* mosquitoes, and one pool, YAP-13-2147, was composed of male *Ae. hensilli* (Table 2). Of 13-positive female pools, 6 pools were composed entirely of *Ae. hensilli*, 6 pools were composed entirely of *Ae. aegypti*, and 1 virus-positive pool, YAP-13-2024, contained two damaged specimens that were identified as *Ae. (Stg.)* spp. All female *Ae. hensilli* virus-positive pools were composed of deplete specimens, whereas three of six positive *Ae. aegypti* pools were composed of engorged specimens (Table 2).

Virus-positive pools were detected at 5 of 14 sample sites (Table 2). Four pools were suspect case-patient residences, and one pool, site 3, was the Police Department and jail located in central Colonia. Virus-positive mosquitoes were collected on multiple days (2–4 days) at three of four case-patient residences (Table 2). Five of six *Ae. aegypti*-positive pools came from a single very productive case-patient residence (site 9). The maximum likelihood estimate^22^ of the IR for *Ae. hensilli* female specimens at virus-positive sites varied from 29 to 127 per 1,000, with an overall IR for all sites over the study period of 38 per 1,000. The IR for *Ae. aegypti* at site 9 was 80 per 1,000, and the overall IR of *Ae. aegypti* at all sites over the study period was 70 per 1,000. Excluding the virus-positive pools composed of engorged females, in which virus may have originated from the fresh blood of viremic humans that had not replicated or disseminated in the mosquito, would result in an IR for *Ae. aegypti* of 36 per 1,000. The 95% confidence interval (95% CI) for difference of proportions^22^ between the overall IR including all female specimens of *Ae. aegypti* and *Ae. hensilli* includes zero (95% CI = −26–105), indicating that the IRs in the two species are not significantly different.

Egg papers placed at four sites, including two case-patient residences (sites 3 and 9), the Environmental Protection Agency, and a forested site, produced only *Ae. hensilli* mosquitoes.

### Sequence characteristics and genetic relationships of the mosquito CHIKV isolates.

**Figure 2.**
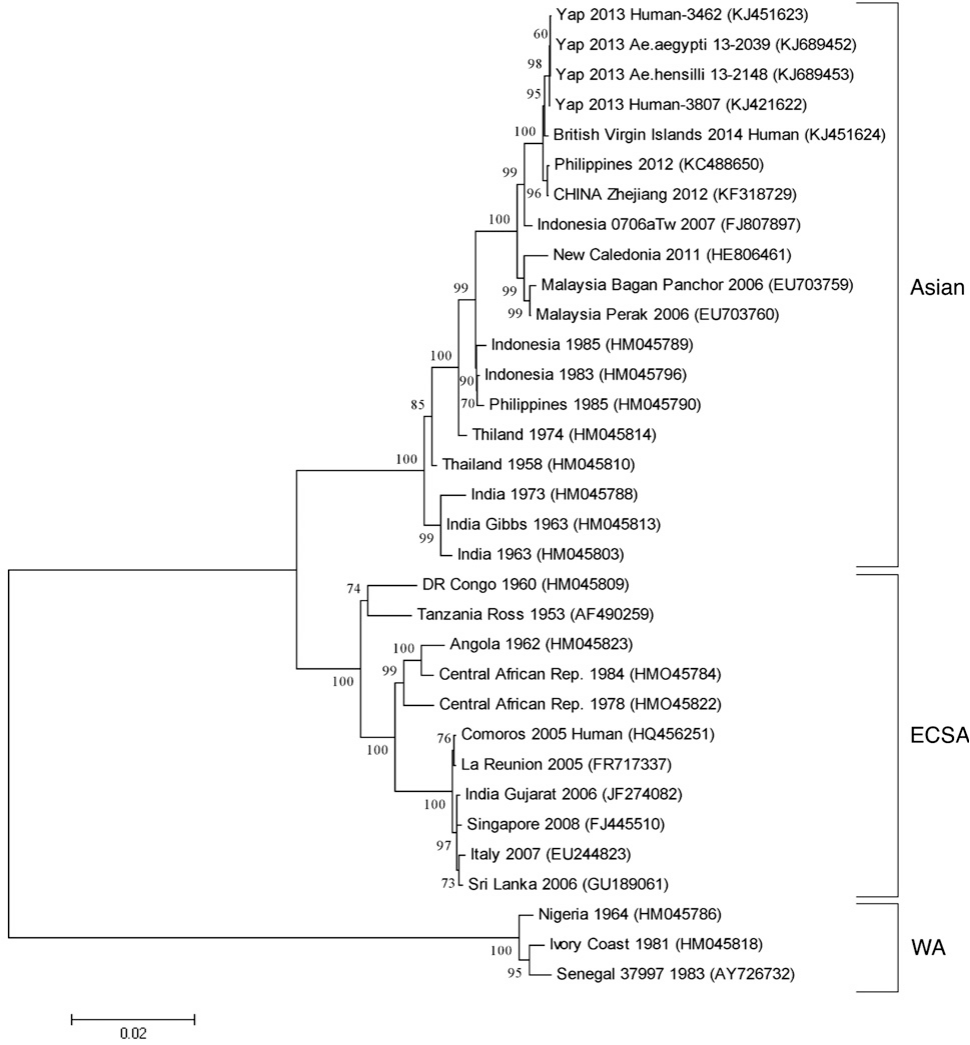
Phylogenic relationship of the mosquito isolates (accession numbers KJ689452 and KJ689453) with 31 representatives of other CHIKV with three major lineages of viruses labeled: Asian, ECSA, and WA. Trees were generated by aligning the available full-length sequences using the maximum likelihood method and the Tamura–Nei model. Bootstrap values were determined using 1,000 replicates, and values are provided at each node. Gaps in the alignment were analyzed by partial deletion, which resulted in 11,244 positions in the final dataset. Accession numbers are listed for each strain.

Full viral genome sequence was obtained from one *Ae. aegypti* (YAP-13-2039) and one *Ae. hensilli* (YAP-13-2148) mosquito pool (Table 2). Each sequence contained 12,043 nucleotides, non-structural and structural open reading frames that encode the alphavirus genes, an OPAL stop codon at the nsP3–nsP4 junction, and untranslated regions at the 5′ end (76 nucleotides), the intergenic junction region (65 nucleotides), and the 3′ end (741 nucleotides; including a poly A tail). The two mosquito pool isolates are very closely related to each other and two CHIKV isolates obtained from human cases during the Yap outbreak.^6^ Both human and mosquito isolates are members of the Asian genotype and very closely related to the strain currently circulating in the Caribbean (Figure 2

). As members of the Asian genotype, the Yap CHIKV isolates lack the alanine to valine substitution, E1-A226V, found in some ECSA strains, which has been associated with increased virus infectivity, dissemination, and transmission in *Ae. albopictus*.^9^,^10^,^23^ Only four changes were observed between the mosquito and human isolates from Yap: an R (A or G) at nucleotide position 764, a C–T change at nucleotide position 4,365 (only between human isolates), a T–C change at nucleotide position 4,540, and an R at nucleotide position 5,302 (Table 3). Three of these changes result in amino acid changes in either the nsP1 or the nsP3 gene, and these genes have functions in the capping of messenger RNA^24^,^25^ and a regulatory role in RNA synthesis, respectively.^2^,^26^

## Discussion

Epidemic transmission of CHIKV has been primarily associated with two species of *Ae. (Stg.)*: *Ae. aegypti* and *Ae. albopictus*. *Ae. aegypti* is an African species, and *Ae. albopictus* is an Asian species. Both species are successful colonizers and presently display greatly expanded distributions because of human commerce.

We detected two species of *Ae. (Stg.)* on Yap: the introduced species *Ae. aegypti* and the native species *Ae. hensilli*. *Ae. albopictus* was not present in our collections or those of three previous surveys (Savage HM and Godsey M, unpublished data).^27^,^28^ *Ae. hensilli* is one of the most abundant species on Yap and common in all areas of the island, except the port area (Savage HM and Godsey M, unpublished data).^27^ Larvae of this species occur in both natural and manmade habitats throughout the island,^27^,^28^ including coconut shells and husks, which are common in natural areas and very abundant around homes, where coconut is consumed as a principal food item and coconut husks are stored to make fiber and use as firewood. In contrast, *Ae. aegypti* apparently did not successfully colonize Yap during World War II^14^ and was first detected but rare in 1995 during a survey associated with an outbreak of dengue-4 virus.^27^ Currently, *Ae. aegypti* has a patchy distribution on Yap island, being common in Colonia, the port area, and nearby dense residential areas of northern Rull and less common in other parts of the island; however, *Ae. aegypti* is occasionally present in rural communities, where piped drinking water is not available and/or 55-gallon barrels are abundant (Savage HM and Godsey M, unpublished data).^27^ The only other species of *Ae. (Stg.)* on Yap, *Ae. maehleri* Bohart, is not readily detected in adult collections.^27^,^28^ In the aquatic stages, *Ae. maehleri* is strongly associated with pitcher plants^14^ but does occur in manmade habitats, particularly at residences surrounded by native vegetation.^27^,^28^

Thirteen mosquito pools yielded viable CHIKV, and one additional RNA-positive pool was detected by real-time RT-PCR (Table 2). Six pools were composed entirely of female *Ae. hensilli*, and one pool was composed of four male *Ae. hensilli* specimens. Six positive pools were composed entirely of *Ae. aegypti* females (Table 2). One virus-positive pool contained two badly damaged specimens that were identified as *Ae. (Stg.)* spp. IRs for all female specimens of *Ae. hensilli* and *Ae. aegypti* were not significantly different (*P* ≤ 0.05). Virus-positive pools were detected at 5 of 14 sampled sites (Table 2). Four sites were suspect case-patient residences, and one site, site 3, was the Police Department and jail located in central Colonia. Five of six (83%) *Ae. aegypti* isolates came from a single productive case-patient residence, and three of six positive *Ae. aegypti* pools were composed of engorged specimens. Virus-positive mosquitoes were collected on multiple (2–4) days at three of four case-patient residences (Table 2). Detection of virus on multiple days at case-patient residences and in engorged specimens is indicative of continued transmission at these case-patients' homes. Bed nets were provided to hospitalized patients to limit transmission within the wards; however, bed nets and insect repellents were rare in homes and in very limited supply for outpatients from the hospital, making transmission from viremic outpatients to other residents of virus-positive homes likely. Although the entomological team did not attempt to document continued transmission within a household, we did observe instances where a hospital-identified case-patient was ill but recovering, whereas a different family member was severely ill with symptoms consistent with CHIKV.

The data from case-patient residence site 9, where five of six *Ae. aegypti*-positive pools were identified and no *Ae. hensilli* were collected, show that *Ae. aegypti* is an efficient vector as expected. However, the greater number of sites that yielded virus in *Ae. hensilli* and the much wider distribution and greater abundance of *Ae. hensilli* on Yap suggest that *Ae. hensilli* was a more important vector on Yap than *Ae. aegypti*. The epidemiological data also support this contention. Human CHIKV cases were reported from all municipalities on Yap, indicating that the vector had a wide distribution, and the highest attack rate was observed in Gagil (Pastula D and Hancock WT, personal communication), a rural municipality where *Ae. hensilli* is abundant and *Ae. aegypti* is limited in distribution. Virus-positive pools of *Ae. hensilli* were also detected at the jail in central Colonia (site 3) and a residential property in the surrounding urbanized area (site 7), two sites where the ecology and larvae habitats should have been associated with greater *Ae. aegypti* abundance.

Concurrent with the CHIKV outbreak on Yap, human suspect case-patients were also reported from four islands of Yap State: Fais, Eauripik, Ulithi Atoll, and Ifalik Atoll (Pastula D and Hancock WT, personal communication). Although entomological surveillance was not conducted on these islands during the CHIKV outbreak of 2013, previous entomological surveys documented that *Ae. hensilli* was very common and that *Ae. aegypti* and *Ae. albopictus* were both absent on Fais (Aure F, unpublished data), Eauripik,^27^ and Ulithi and Ifalik Atolls.^29^

Previous studies have implicated *Ae. hensilli* as an epidemic vector of dengue viruses on Yap and Palau based on distribution, abundance, and positive association with risk factors for human disease, despite the absence of virus isolations from field-collected specimens.^27^,^30^ Similarly, *Ae. hensilli* was implicated as an epidemic vector of Zika virus on Yap in 2007 based on abundance and distribution without the support of virus isolations from field-collected specimens.^31^ This report is the first time that *Ae. hensilli* has been incriminated as a vector based on multiple virus isolates from field-collected specimens. Recently, laboratory studies have provided additional support for the vector status of *Ae. hensilli* for both CHIKV and Zika virus and weaker support for its role in dengue virus transmission.^28^

Our detection of CHIKV in one pool composed of four male *Ae. hensilli* was surprising. Previous vector competence experiments and testing of field-collected specimens have provided mixed results, with most studies reporting the absence of vertical transmission^8^,^32^,^33^ but others supporting its occurrence.^34^,^35^ Recent experimental work with *Ae. albopictus* indicates that vertical transmission of CHIKV is rare and limited to the progeny of older females, because Bellini and others^35^ detected virus-positive progeny at low rates (4.3 per 1,000) and only in progeny of the second gonotropic cycle. The epidemiological significance of vertical transmission is uncertain but coupled with venereal transmission, which has been shown in the laboratory for *Ae. aegypti* and CHIKV,^36^ offers a possible secondary mechanism of virus maintenance in the vector.

The sequences for the virus isolates from mosquitoes and humans from Yap are very similar, and all are members of the Asia clade of CHIKV (Figure 2). The Yap strains are most closely related to a human isolate from the British Virgin Islands.^6^ The Yap–British Virgin Islands clade is the sister group to a lineage including two viruses isolated during CHIKV activity in 2012 in Asia: one reported from the Philippines and one reported from a sailor in a Chinese port. This suggests that the CHIKV outbreaks in the Pacific and Caribbean are parts of one larger outbreak and that the virus associated with both outbreaks originated in Asia.

Incrimination of *Ae. hensilli* as an epidemic vector of CHIKV in Yap based on multiple virus isolations from field-collected specimens combined with the established vector status of *Ae. albopictus* suggest that other members of the Scutellaris group of *Ae. (Stg.)* could also serve as vectors of CHIKV, including species occurring on remote islands, where the traditional epidemic vectors *Ae. aegypti* and *Ae. albopictus* are absent or limited in distribution. The implication of a number of species of the Scutellaris group as epidemic vectors of dengue viruses^27^ and the implication of *Ae. hensilli* as an epidemic vector of Zika virus on Yap^28^,^31^ further show the ability of Scutellaris group mosquitoes to vector a variety of viruses in different families. However, CHIKV has been isolated from mosquitoes in several subgenera of *Aedes* as well as the genus *Mansonia*,^1^ and additional taxa may play a role in the transmission of CHIKV as it is introduced and becomes established in new geographic regions.

## Figures and Tables

**Table 1:** Number (percentage) of adult mosquito taxa collected with BG-Sentinel traps and aspirators on Yap during an outbreak of CHIKV (November 13–18, 2013)

Taxa	Female, *n* (%)	Male, *n* (%)	Total, *n* (%)
*Ae. (Aedimorphus) vexans nocturnus*	7 (1.0)		7 (0.4)
*Ae. (Finlaya)* near hui	2 (0.3)	1 (0.1)	3 (0.2)
*Ae. (Stegomyia) aegypti*	93 (13.3)	100 (10.4)	193 (11.6)
*Ae. (Stegomyia) hensilli*	183 (26.2)	304 (31.6)	487 (29.3)
*Ae. (Stegomyia)* spp.	7 (1.0)		7 (0.4)
*Cx. (Cux.) quinquefasciatus*	405 (58.0)	557 (57.9)	962 (58.0)
*Cx. (Lophoceraomyia) gossi*	1 (0.1)		1 (< 0.1)
Total	698 (42.0)	962 (58.0)	1,660

**Table 2:** Information on CHIKV-positive mosquito pools by real-time RT-PCR collected on Yap during an epidemic (November of 2013)

Site number	Municipality	Collection date	Collection method	Species	Sex	Pool composition		Pool number	GenBank accession
						Engorged	Depleted		
13-2	Tomil	November 14	BG trap	*Ae. hensilli*	Female	0	14	YAP-13-2055*	
13-2	Tomil	November 15	BG trap	*Ae. hensilli*	Female	0	8	YAP-13-2081*	
13-2	Tomil	November 16	BG trap	*Ae. hensilli*	Female	0	12	YAP-13-2107*	
13-3†	Weloy (Colonia)	November 13	BG trap	*Ae. hensilli*	Female	0	3	YAP-13-2018*	
13-6	Rull	November 14	BG trap	*Ae. (Stegomyia)* spp.	Female	0	2	YAP-13-2024*	
13-7	Rull	November 17	BG trap	*Ae. hensilli*	Male	0	4	YAP-13-2147*	
13-7	Rull	November 17	BG trap	*Ae. hensilli*	Female	0	6	YAP-13-2148*	KJ689453
13-7	Rull	November 17	BG trap	*Ae. aegypti*	Female	0	1	YAP-13-2149*	
13-7	Rull	November 18	BG trap	*Ae. hensilli*	Female	0	12	YAP-13-2193*	
13-9	Rull	November 14	BG trap	*Ae. aegypti*	Female	1	0	YAP-13-2039*	KJ689452
13-9	Rull	November 15	Aspirator	*Ae. aegypti*	Female	5	0	YAP-13-2096*	
13-9	Rull	November 16	BG trap	*Ae. aegypti*	Female	0	7	YAP-13-2130*	
13-9	Rull	November 17	Aspirator	*Ae. aegypti*	Female	2	0	YAP-13-2169	
13-9	Rull	November 17	Aspirator	*Ae. aegypti*	Female	0	7	YAP-13-2170*	

* Thirteen mosquito pools yielded viable virus.

† All positive sites were suspect case-patient residences except site 13-3, which was the Division of Public Safety (Police Department and jail).

**Table 3:** Genome differences observed between the CHIKV isolates from Yap in 2013.

	Nucleotide location* (gene)			
Strain	764 (nsP1)	4,365 (nsP3)	4,540 (nsP3)	5,302 (nsP3)
*Ae. hensilli* 13-2148	R†	C	T	R
*Ae. aegypti* 13-2039	R	C	T	A
Human 13-3807	G	C	C	A
Human 13-3462	G	T	T	A
Amino acid change	Gly→Arg	Thr→Ile	Silent	Ile→Met

* From the start of the genome.

† A or G.
